# 

**Published:** 1843-02

**Authors:** 


### WESTERN JOURNAL

OF

### MEDICINE AND SURGERY.

### EDITORS.

---

DANIEL DRAKE, M. D.,

PROFESSOR OF PATHOLOGICAL ANATOMY AND CLINICAL MEDICINE

IN THE MEDICAL INSTITUTE OF LOUISVILLE:

LUNSFORD P. YANDELL, M. D.,

PROFESSOR OF CHEMISTRY AND PHARMACY IN THE SAME:

THOMAS W. COLESCOTT, M. D.

### RECEIPTS OF THE MEDICAL JOURNAL.

FOR THE MONTHS OF JANUARY AND FEBRUARY.

| | |
|---|---|
| Dr. S. C. Eartholomew, Greensburg, Ia., - - - - | $7 00 |
| C. H. Preston, Paris, O., - - - - - - | 3 00 |
| P. Q. Stryker, Rockville, Ia., - - - - - | 10 00 |
| A. H. King, Carthage, Tenn., - - - - - | 10 00 |
| Philip Winfree, New River, La., - - - - | 5 00 |
| L. M. N. Cook, Silver Spring, Tenn., - - - | 10 00 |
| J. F. Fleming, Elizaville, Ky., - - - - - | 5 00 |
| John Carriss, Salem, O., - - - - - - | 5 00 |
| T. S. Davidson, Perryville, Ia., - - - - - | 5 00 |
| W. B. Langly, White Hall, Tenn., - - - - | 10 00 |
| D. M. Porter, Sharon, Miss. - - - - - | 5 00 |
| J. J. Beaty, Gilbertsboro, Ala., - - - - - | 5 00 |
| H. Slaughter, Elizabethtown, Ky., - - - - | 5 00 |
| C. S. Bowen, Chulahoma, Miss., - - - - - | 2 50 |
| David M'Call, Rome, Tenn., - - - - - | 10 00 |
| C. T. Pomeroy, Bunkeye, O., - - - - - | 5 00 |
| W. B. Holmes, Granville, Teen., - - - - | 5 00 |
| E. H. Watts, Gainesville, Ala., (discount $2 50—postage 50,) - - - | 10 00 |
| J. F. Souell, Athens, Ala., (discount $1 50,) - - - - - - | 5 00 |
| J. W. Beauchamp, Edmonton, Ky., - - - - | 5 00 |
| J. R. Desha, Georgetewn, Ky., - - - - - | 5 00 |
| J. Harris, Salem, O., - - - - - - - | 5 00 |
| B. Albertson, Canton, Ia., - - - - - - | 6 00 |

